# miRNA-328-3p regulates ZO-1 expression and inhibits PEDV proliferation via the PLC-β1-PKC pathway

**DOI:** 10.1371/journal.pone.0316074

**Published:** 2025-01-03

**Authors:** Han Zhao, Jiayu Zhang, Dengliang Li, Zhanding Cui, Jiuyuan Liu, Di Bao, Yiming Wei, Xinyue Zhang, Zhimin Wu, Tianqi Zhang, Kai Wang, Shushuai Yi, Wei Lian, Guixue Hu

**Affiliations:** 1 College of Veterinary Medicine, Jilin Agricultural University, Changchun, China; 2 Changchun Chengkai Agricultural Investment Livestock Development Co.,Ltd.,Changchun, China; 3 College of Agriculture, Tongren Polytechnic College, Tongren, Guizhou, Cnina; 4 Department of Veterinary Medicine, Key Laboratory of Preventive Veterinary Medicine, Animal Science College, Hebei North University, Zhangjiakou, Hebei, China; 5 Suzhou Jinweizhi Biotechnology Co.,Ltd, Suzhou, China; 6 Chengdu Zhentuo Pharmaceutical Technology Co., Ltd, Chendu, China; 7 College of Veterinary Medicine, Jilin Agricultural Science and Technology University, Jilin, China; 8 Jilin ZhengYe Biological Products Co.,Ltd., Jilin, China; Louisiana State University, UNITED STATES OF AMERICA

## Abstract

Porcine epidemic diarrhea virus (PEDV) is a significant pathogen affecting swine, causing severe economic losses worldwide. This study explores the regulatory role of miRNA-328-3p to ZO-1 expression and its impact on PEDV proliferation via the PLC-β1-PKC pathway in IPEC-J2 cells. We found that miRNA-328-3p can target ZO-1, influencing its expression and subsequently affecting the integrity of tight junctions in the cells. Overexpression of PLC-β1, combined with miRNA-328-3p silencing, enhanced ZO-1 expression, while PLC-β1 knockdown combined with miRNA-328-3p overexpression inhibited ZO-1 expression. Furthermore, PLC-β1 overexpression increased both viral genome expression and PEDV titers, whereas its silencing had the opposite effect. Notably, our data indicated a negative correlation between PLC-β1 and PKC expression, and PKC silencing attenuated the upregulatory effect of PLC-β1 on ZO-1. These findings suggest that PLC-β1 modulates ZO-1 expression through the PKC pathway, providing new insights into the molecular mechanisms of PEDV infection and potential therapeutic targets.

## Introduction

Porcine Epidemic Diarrhea Virus (PEDV) was first identified in the early 1970s in the UK and Belgium, with its initial isolation occurring in Belgium in 1977 [1]. It is classified as a member of the Coronaviridae family [2]. Throughout the 1980s and 1990s, PEDV emerged as a severe epidemic infectious disease in Japan and Korea [3]. Despite the widespread use of PEDV vaccines, porcine Epidemic Diarrhea (PED) continues to be prevalent worldwide [4]. Understanding the pathogenic mechanisms underlying intestinal damage caused by PEDV infection and exploring effective prevention and control measures are urgently needed.

Previous studies have shown that tight junction (TJ) proteins are closely related to PEDV infection, possibly due to their role in maintaining and repairing the intestinal mucosal barrier [5,6]. The integrity of this barrier depends on the connection of tight junction proteins, including zonula occludens-1 (ZO-1), ZO-2, and ZO-3 [7]. Researchers have discovered that PEDV can promote infection and spread within animals by disrupting the nasal epithelial barrier [8]. Increased permeability resulting from disrupting the intestinal barrier can enhance the infectivity of PEDV [9,10]. PEDV infection of epithelial cells leads to TJ disruption and the redistribution of tight junction proteins within cells [11,12]. Additionally, the TJ protein occludin has been identified as crucial for PEDV invasion, potentially aiding PEDV internalization [13,14]. However, the roles of other TJ proteins in PEDV infection remain unclear.

In our previous research, we found that miRNA-328-3p in exosomes could target ZO-3 and inhibit PEDV proliferation [15]. MicroRNAs (miRNAs) are small non-coding RNAs that regulate gene expression by degrading mRNA or inhibiting translation. miRNAs primarily target mRNA by pairing with the 3′ untranslated region (UTR), thereby directly regulating post-transcriptional repression [16]. A recent study found that miR-let-7e derived from milk exosomes could inhibit PEDV replication by targeting the PEDV N protein. In this study, we found a connection between miRNA-328-3p and ZO-1, which related to PEDV infection [17]. We discovered that miRNA-328-3p might influence ZO-1 expression through the PLC-β1-PKC pathway, thereby inhibiting PEDV proliferation. Elucidating this new mechanism of miRNA-328-3p against PEDV infection is of significant practical and economic importance for controlling the spread of PEDV.

## Methods and materials

### Cells and strain

Intestinal Porcine Epithelial Cell line-J2 (IPEC-J2) and the classic PEDV CV777 strain were preserved in our laboratory.

## Plasmid construction and antibodies

miRNA-328-3p was designed and synthesized with Cy3 labeling by Sangon Biotech (Shanghai, China) Co,. Ltd. The miRNA-328-3p mimics, non-labeled miRNA-inhibitor, and NC control were also obtained from Sangon Biotech Co. The sequences are as follows: miRNA-328-3p mimics: 5′-CUGGCCCUCUCUGCC CUUCCG-3′; miRNA-328-3p inhibitor: 5′-CGGAAGGGCAGAGAGGGCCAG-3′; NC: 5′-UUGUACUACACAAAAGUACUG-3′; PLC-β1 and PKC genes were cloned into the pCMV-HA vector (Shanghai Zeye Biotechnology Co,. Ltd) via enzymatic ligation to obtain PCMV-PLC-β1 and PCMV-PKC plasmids, respectively. The following antibodies were used for Western blotting: rabbit anti-ZO-1 polyclonal antibody (Invitrogen); rabbit anti-PLC-β1 polyclonal antibody (Affinity); mouse anti-GAPDH polyclonal antibody (Abcam); rabbit anti-PKC polyclonal antibody (Invitrogen); HRP-conjugated goat anti-rabbit IgG (Servicebio); HRP-conjugated goat anti-mouse IgG (Servicebio). The following antibodies were used for Immunofluorescence staining and confocal microscopy: abbit anti-ZO-1 polyclonal antibody (Invitrogen); PEDV N-protein monoclonal antibodies (LLC dba Medgene Labs); Cy3-conjugated goat anti-rabbit IgG (Servicebio); FITC-goat anti-mouse IgG (Servicebio). For indirect immunofluorescence analysis (IFA), rabbit anti-ZO-1 polyclonal antibody (Invitrogen) and Cy3-conjugated goat anti-rabbit IgG (Servicebio) were used.

### RNA extraction and quantitative Real-Time RT-PCR analysis

Cells were harvested at different time points post-infection of PEDV or as required by the experiment. After washing three times with pre-chilled PBS, total RNA was extracted using an RNA extraction kit (BioFlux Company, Tokyo, Japan) according to the manufacturer’s instructions. cDNA was synthesized using the cDNA first strand synthesis kit (TaKaRa, Dalian, China). qRT-PCR was performed using SYBR® Premix Ex Taq™ II (TaKaRa, Dalian, China). Primer sequences are shown in Table 1. Briefly, the 20 μL reaction system included: ROX Reference Dye II (50×) 0.2 μL, SYBR Premix Ex Taq II (2×) 10 μL, forward primer 1 μL, reverse primer 1 μL, cDNA 2 μL, and H2O 5.8 μL. The reaction conditions were: 95°C for 30 s, 95°C for 5 s, and 60°C for 30 s, with 40 cycles. β-actin expression was measured as an internal control for each sample.

**Table 1:** Primers used in this study.

Gene	Primer	Sequence (5’-3’)
*ZO-1*	sense	CCAGGGAGAGAAGTGCCAGTAGG
	antisense	TTTGGTGGGTTTGGTGGGTTGAC
*PEDV*	sense	CAAGTTCAAGGAACTGCC
	antisense	CATCAACATATGCAGCCTG
*PLC-β1*	sense	GCGCAAAGTAAACGGCAAGA
	antisense	CTGCAGCTTGGGCTTTTCAT
*PKC*	sense	GGACCAAGACTCCCGAAGAG
	antisense	CAGGATCTTCACCGCGTACA
*β-actin*	sense	TGCGGGACATCAAGGAGAAG
	antisense	AGTTGAAGGTGGTCTCGTGG

### Virus titration

Each sample was serially diluted tenfold, and 100 μL of each dilution was added to each well in eight columns of a microtiter plate, followed by 100 μL of DMEM (Gibco) containing 2% FBS (Gibco). Control wells contained virus-free medium, and plates were incubated at 37°C with 5% CO_2_. The Reed-Muench method was used to calculate the 50% tissue culture infective dose (TCID_50_).

### Western blotting

Experiments were performed as previously described [18]. Protein concentrations were determined using a BCA protein assay kit (Biouniquer Technology Co., LTD, Nanjing, China), and 20 μg of protein lysate from each sample was analyzed. Proteins were separated by SDS-PAGE and transferred to polyvinylidene fluoride (PVDF, ThermoFisher Scientific) membranes. Non-specific binding sites were blocked with 5% non-fat milk for 1 hour. Blots were incubated with primary and secondary antibodies, visualized using an ECL detection kit (Beyotime, Shanghai, China), and quantified using Image Tool 3.0 software.

### Immunofluorescence staining

For confocal microscopy, performed as previously described [19]. Cells were fixed with 80% cold acetone according to previous IFA conditions and observed under a Leica DMI6000 microscope.

For confocal microscopy, cells were fixed with 4% paraformaldehyde at room tem perature for 30 min. The samples were then blocked and permeabilized in PBS containing 5% horse serum and 0.1% Triton X-100 for 1 h at room temperature and incubated with primary antibodies(rabbit anti-ZO-1 polyclonal antibody; PEDV N-protein monoclonal antibodies) at 4 °C overnight, followed by incubation with fluorescence-conjugated secondary anti bodies(Cy3-conjugated goat anti-rabbit IgG; FITC-goat anti-mouse IgG) at room temperature for 1 h. Nuclei were counterstained with 4,6-diamidino-2-phenylindole (DAPI). Images were taken using a Zeiss LSM 800 confocal microscope.

### Cell transfection

Cells were transfected using Lipofectamine 3000 (Invitrogen) when they reached 80% confluence in 24-well plates. The procedure involved adding DMEM with 10% FBS to six-well plates, replacing the medium with DMEM when cell coverage reached 70%. miRNA, inhibitor (50 nM), siRNA (2 μg), and Lipofectamine 3000 were mixed with an equal volume of DMEM (500 μL) in centrifuge tubes and incubated at 37°C with 5% CO_2_ for 24 hours for subsequent experiments.

### RNA interference

IPEC-J2 cells were transfected when cell density reached 70–80% in six-well plates. Lipofectamine 3000 (5 μL) was mixed with 125 μL Opti-MEM medium (Invitrogen) in one EP tube, and 125 μL Opti-MEM with siRNA was gently mixed in another tube. After a 5-minute rest, the solutions were combined and incubated at room temperature for 15 minutes. mRNA or protein expression levels were detected 24 hours later. The siRNA sequences were: PLCβ1-siRNA: 5′-CACCAUGACAACUGAAAUATT-3′′; PLCβ1 Negative control: 5′-UUCUCCGAACGUGUCACGUTT-3′; PKC-siRNA: 5′-CAGAAAGCAGAUUG UUCUUUGUCAU-3′; PKC Negative control: 5′-AUGACAAAGAACAAUCUGCU UUCUG-3′; ZO-1-siRNA: 5′- GGGACAAGAUGAAGUACCAGAAAUA-3′; ZO-1 Negative control: 5′-UAUUUCUGGUACUUCAUCUUGUCCC-3′; siRNA was synthesized by Shanghai GenePharma Co., Ltd.

### Proteomics

Approximately 2 μg of peptide from each sample was separated and analyzed using nano-ultra-performance liquid chromatography (nano-UPLC, EASY-nLC1200) coupled with Q-Exactive mass spectrometry (Thermo Finnigan). The separation was conducted using a reversed-phase column (100 μm ID × 15 cm, Reprosil-Pur 120 C18-AQ, 1.9 μm, Dr. Math). The mobile phases used were H_2_O with 0.1% formic acid (FA) and 2% acetonitrile (ACN) for phase A, and 80% ACN with 0.1% FA for phase B. Sample separation was executed with a 120-minute gradient at a 300 nL/min flow rate: 8–30% B for 92 minutes, 30–40% B for 20 minutes, 40–100% B for 2 minutes, 100% B for 2 minutes, 100–2% B for 2 minutes, and 2% B for 2 minutes.

Data-dependent acquisition was performed in profile and positive mode using the Orbitrap analyzer with a resolution of 70,000 (@200 m/z) and an m/z range of 350–1600 for MS1. For MS2, the resolution was set to 17,500 with a dynamic first mass. The automatic gain control (AGC) target for MS1 was set to 3.0 × 10^6^ with a maximum injection time (IT) of 50 ms, and 5.0 × 10^4^ for MS2 with a maximum IT of 100 ms. The top 20 most intense ions were fragmented by higher-energy collisional dissociation (HCD) with a normalized collision energy (NCE) of 27% and an isolation window of 2 m/z. The dynamic exclusion time window was set to 30 seconds. Raw MS files were processed with MaxQuant (Version 1.5.6.0). The protein sequence database (Uniprot_organism_2016_09) was downloaded from UNIPROT. This database and its reverse decoy were then searched against using MaxQuant software.

### Statistical analysis

All data in the experimental results were represented as mean±standard error. Statistical comparisons between different groups were performed using an ordinary tow-way ANOVA followed by post Sikad’s multiple-comparison test or by an unpaired two-tailed Student’ t test for two groups using GraphPad Prism (GraphPad Software). When the p-value was less than 0.05, it was considered statistically significant (* p<0.05, ** p<0.01, ns represents no significant difference).

## Results

### ZO-1 involvement in PEDV infection

**Fig 1:**
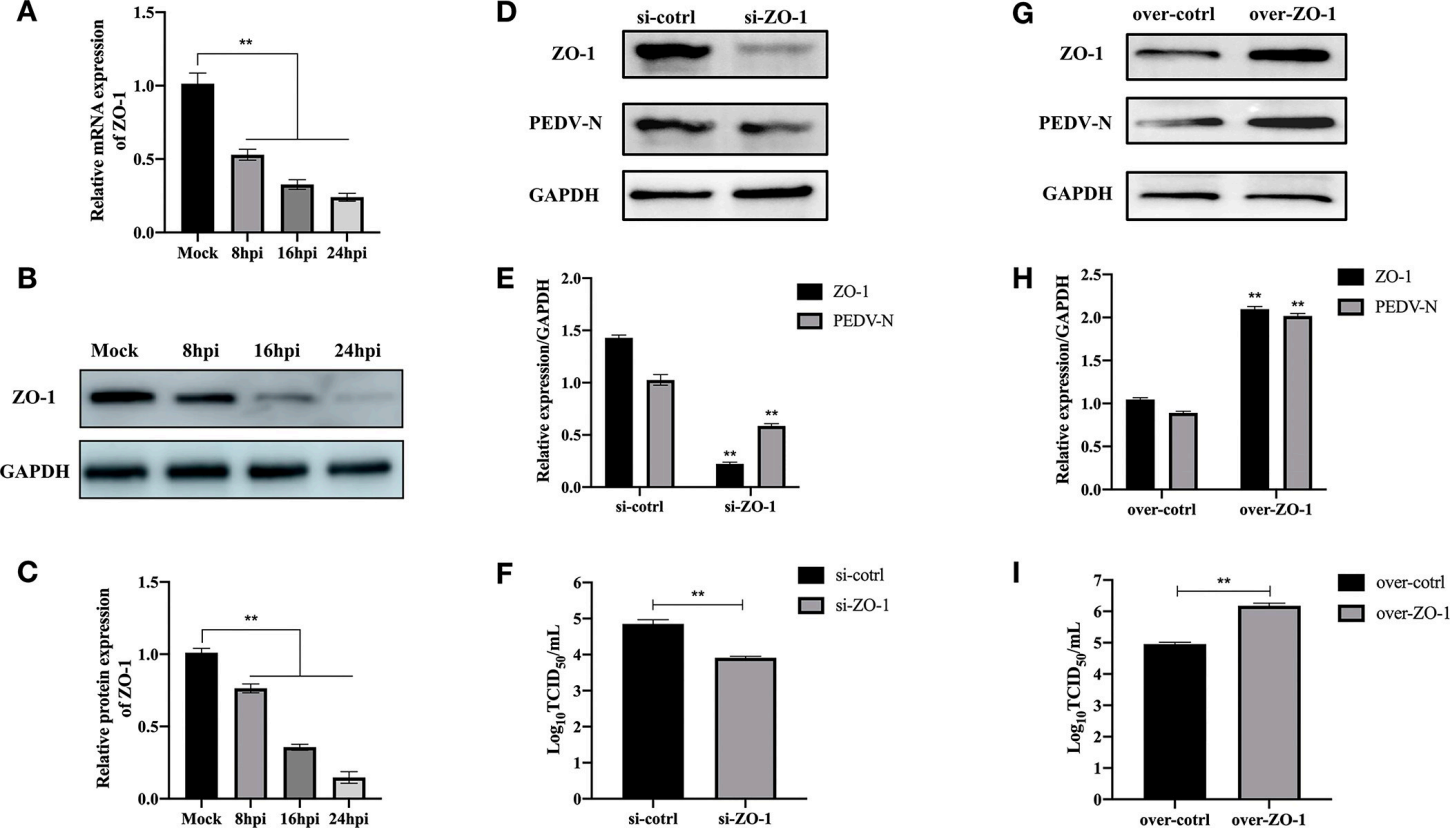
PEDV infection reduces ZO-1 expression and ZO-1 enhances PEDV proliferation. **(A)** PEDV infection decreases ZO-1 mRNA expression levels. **(B)** PEDV infection decreases ZO-1 protein expression levels. **(C)** Densitometric analysis of the bands from the Western blot (WB) shown in Fig 1B. Error bars represent the mean and standard deviation (SD) from three independent WB experiments. **(D)** Knockdown of ZO-1 reduces the expression levels of the PEDV-N protein. **(E)** Effect of ZO-1 knockdown on the mRNA levels of ZO-1 and PEDV-N proteins. **(F)** Effect of ZO-1 knockdown on PEDV viral titers. **(G)** Overexpression of ZO-1 enhances the expression levels of the PEDV-N protein. **(H)** Overexpression of ZO-1 enhances the mRNA levels of ZO-1 and PEDV-N proteins. **(I)** Overexpression of ZO-1 increases PEDV viral titers. All experiments were repeated three times. Error bars represent the mean and standard deviation. **, p<0.01.

TJ proteins are closely related to the proliferation of PEDV. We found that the TJ protein family member ZO-1 was significantly involved in PEDV proliferation. PEDV infection decreases ZO-1 expression time-dependent (Fig 1A–1C). Moreover, the reduction of ZO-1 expression affects PEDV proliferation, as silencing ZO-1 significantly decreases the expression levels of the PEDV-N protein (p<0.01; Figs 1D, 1E and S1). Correspondingly, the viral titer of PEDV also shows a significant reduction (p<0.01; Fig 1F). Conversely, overexpression of ZO-1 in IPEC-J2 cells significantly increases the expression levels of the PEDV-N protein (p<0.01; Figs 1G, 1H and S1). Unsurprisingly, this is accompanied by an increase in the viral titer of PEDV (p<0.01; Fig 1I).

### miRNA-328-3p negatively regulates ZO-1 and affects PEDV proliferation

**Fig 2:**
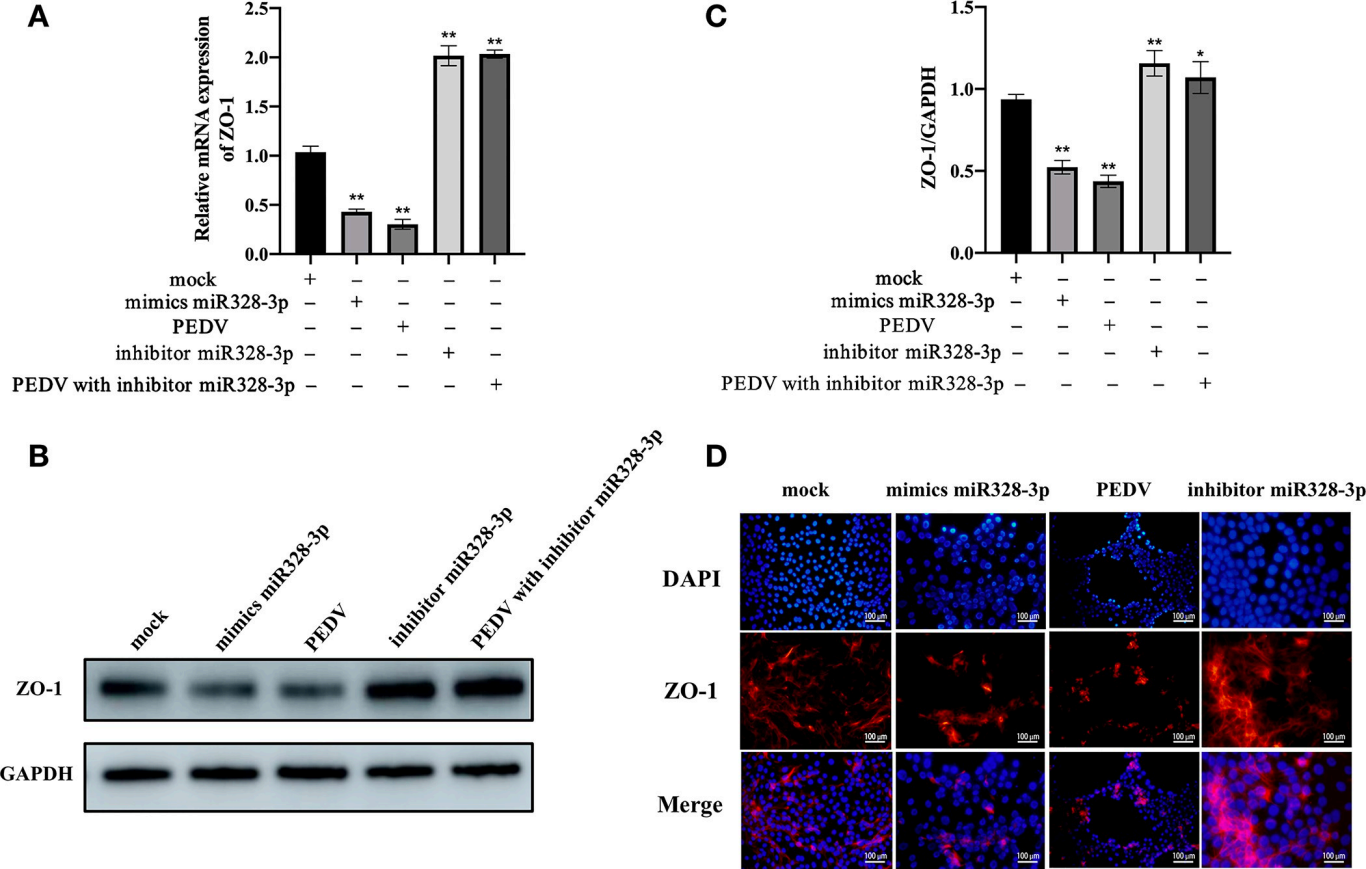
PEDV infection and miRNA-328-3p both reduce ZO-1 expression levels. (A) miRNA-328-3p and PEDV affect the mRNA expression levels of ZO-1. (B) miRNA-328-3p and PEDV influence the protein expression levels of ZO-1. (C) Densitometric analysis of bands from Fig 2B. Error bars represent the standard deviation from three independent WB experiments. (D) IFA results showed the effects of miRNA-328-3p and PEDV on ZO-1 expression. All experiments were performed in triplicate. Error bars represent the mean ± standard deviation. *, p<0.05; **, p<0.01.

Given our previous findings that miRNA-328-3p is associated with ZO-3 expression levels [15], we further explored the relationship between miRNA-328-3p and ZO-1 in subsequent experiments. Interestingly, miRNA-328-3p was found to negatively regulate ZO-1 expression. The mimics of miRNA-328-3p, as well as PEDV infection, significantly reduced ZO-1 expression levels (p<0.01; Fig 2A). Conversely, the miRNA-328-3p inhibitor significantly increased ZO-1 expression levels (p<0.01; Fig 2A). Similar results were observed through WB and IFA (Fig 2B and 2D). Additionally, we found that ZO-1 expression levels significantly increased upon PEDV infection with the addition of the miRNA-328-3p inhibitor (p<0.05; Fig 2A–2C). We hypothesize that the changes of ZO-1 expression may be due to the induction of miRNA-328-3p production following PEDV infection.

### miRNA-328-3p negatively regulates PLC-β1

**Fig 3:**
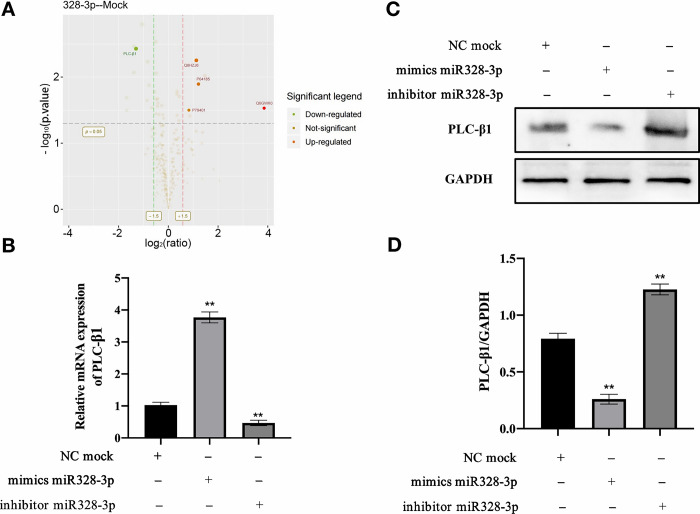
miRNA-328-3p downregulates the expression of PLC-β1. **(A)** The impact of miRNA-328-3p on the proteomics profile of IPEC-J2 cells. Proteomic analysis reveals that miRNA-328-3p downregulates the expression of PLC-β1. **(B)** miRNA-328-3p downregulates the mRNA expression level of PLC-β1. **(C)** miRNA-328-3p downregulates the protein expression level of PLC-β1. **(D)** Densitometric analysis of the Western blot results shown in panel. Error bars represent the standard deviation from three independent Western blot analyses. All experiments were performed in triplicate, except for the proteomic analysis, which was repeated three times for technical validation. **, p < 0.01.

According to our previous data, miRNA-328-3p does not have a direct interaction with ZO-1 mRNA (data not shown). We were curious about how miRNA-328-3p regulates ZO-1 expression. Therefore, we transfected miRNA-328-3p mimics into IPEC-J2 cells and performed proteomic sequencing. We found that miRNA-328-3p downregulates the expression of PLC-β1 (Fig 3A). Validation of PLC-β1 mRNA levels showed a negative correlation with miRNA-328-3p (Fig 3B). Additionally, Western blot analysis confirmed that miRNA-328-3p mimics significantly reduced the expression levels of PLC-β1 protein (p<0.01; Fig 3C and 3D), while miRNA-328-3p inhibitors significantly increased PLC-β1 protein expression levels (p<0.01; Fig 3C and 3D).

### PLC-β1 positively regulates ZO-1 and influences PEDV infection

**Fig 4:**
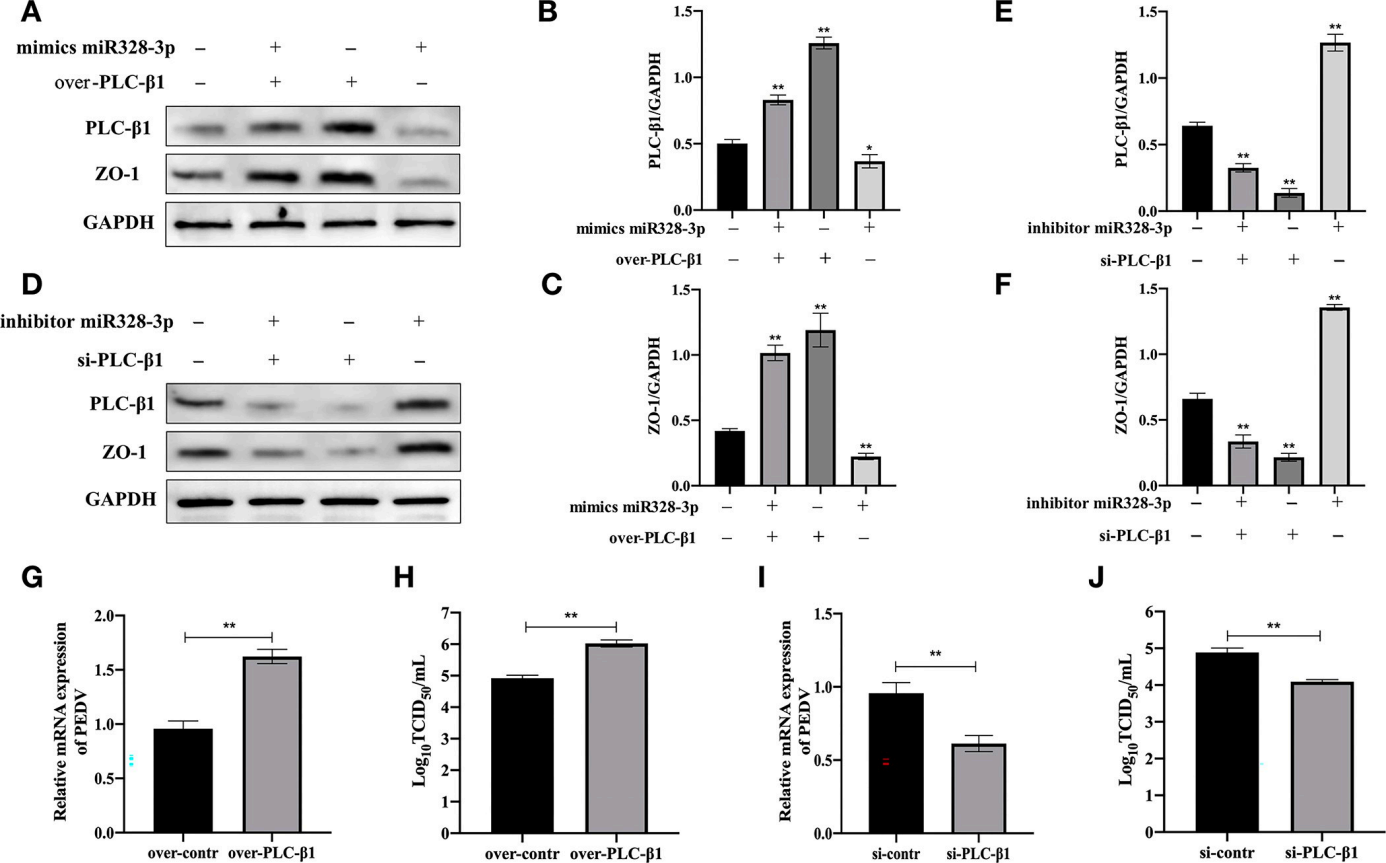
PLC-β1 upregulates ZO-1 expression and promotes PEDV infection. **(A)** Overexpression of PLC-β1 promotes ZO-1 expression and counteracts the negative regulatory effect of miRNA-328-3p on ZO-1. **(B)** Silencing of PLC-β1 inhibits ZO-1 expression and enhances the negative regulatory effect of miRNA-328-3p on ZO-1. **(C)** Densitometric analysis of PLC-β1 protein in Fig 4A. **(D)** Densitometric analysis of ZO-1 protein in Fig 4A. **(E)** Densitometric analysis of PLC-β1 protein in Fig 4B. **(F)** Densitometric analysis of ZO-1 protein in Fig 4B. **(G)** Overexpression of PLC-β1 increases viral genome expression levels. **(H)** Overexpression of PLC-β1 increases PEDV viral titers. **(I)** Silencing of PLC-β1 reduces viral genome expression levels. **(J)** Silencing of PLC-β1 reduces PEDV viral titers.All experiments were repeated three times. In densitometric analyses, error bars represent results from three independent WB images. Error bars represent mean ± standard deviation. *, p<0.05; **, p<0.01.

In subsequent experiments, we further investigated the relationship between PLC-β1 and ZO-1. We found that overexpression of PLC-β1 (p<0.01; Fig 4A and 4C) and miRNA-328-3p mimics both significantly increased the expression of ZO-1 (p<0.01; Fig 4A and 4D). Conversely, silencing PLC-β1 (p<0.01; Fig 4B and 4E) and using miRNA-328-3p inhibitors significantly reduced ZO-1 expression (p<0.01; Fig 4B and 4F). These findings suggest that miRNA-328-3p may regulate ZO-1 expression by negatively modulating PLC-β1. Furthermore, PLC-β1 overexpression enhanced the genomic levels of PEDV and significantly increased viral titers (p<0.01; Fig 4G and 4H). In contrast, silencing PLC-β1 reduced the genomic levels of PEDV and significantly decreased viral titers (p<0.01; Fig 4I and 4J). These results indicate that PLC-β1 may influence PEDV proliferation by positively regulating ZO-1.

### PLC-β1 mediates ZO-1 regulation through the PKC pathway

**Fig 5:**
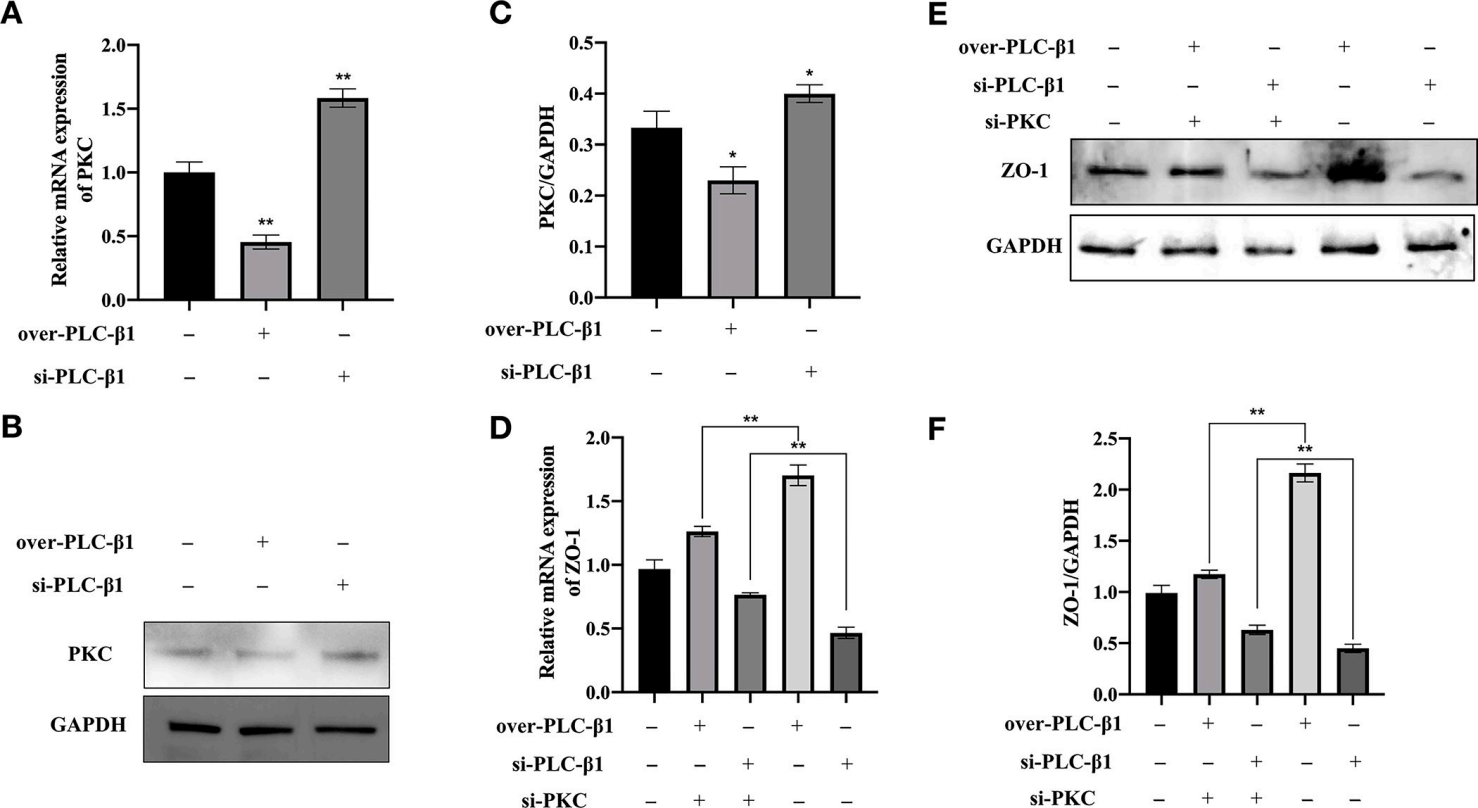
The effect of PLC-β1 on ZO-1 expression is mediated via the PKC pathway. **(A)** Negative correlation between PLC-β1 and PKC mRNA expression. **(B)** Negative correlation between PLC-β1 and PKC protein expression. **(C)** Gray scanning analysis of Fig 5B. Error bars represent results from scanning three independent WB images. **(D)** Silencing PKC reduces the upregulatory effect of PLC-β1 on ZO-1 mRNA expression. **(E)** Silencing PKC reduces the upregulatory effect of PLC-β1 on ZO-1 protein expression. **(F)** Gray scanning analysis of Fig 5E. All experiments were repeated three times. Error bars represent mean ± standard deviation. *, p<0.05; **, p<0.01.

Previous studies indicated that PLC-β1 does not directly impact ZO-1 expression. However, PLC-β1 may influence ZO-1 expression through the PKC pathway. Subsequently, we observed a negative correlation between PLC-β1 and PKC expression (p<0.05; Fig 5A–5C). Additionally, silencing PKC attenuated the upregulatory effect of PLC-β1 on ZO-1 expression (p<0.01; Fig 5D–5F). These findings suggest that PLC-β1 may regulate ZO-1 expression via the PKC pathway.

## Discussion

TJs are essential for maintaining the integrity of the intestinal barrier, which in turn ensures normal intestinal function [20]. The compromise of the intestinal barrier is associated with various bacterial and viral infections. For PEDV, the intestine is a significant target organ, and infection by PEDV increases intestinal permeability, leading to clinical symptoms such as diarrhea. TJ proteins link with the actin-based cytoskeleton through adapter and scaffold molecules, and the interactions of these proteins are critical for normal intestinal barrier function [20]. The ZO-1 scaffold protein family is the most crucial multi-domain protein [21]. ZO-1 plays a vital role in coordinating TJ formation and cell polarization, as it establishes connections between most transmembrane TJ proteins (i.e., claudins, TAMP, and JAM) and cytoskeletal components [22]. Previous studies have largely linked viral infection-induced intestinal barrier damage with TJ proteins [23].

In our study, we first identified the involvement of ZO-1 in PEDV infection. Previous research indicated that PEDV infection of nasal epithelial cells downregulates ZO-1 expression [8]. Additionally, the downregulation of ZO-1 signifies damage to the endothelial barrier and significantly affects its permeability [24]. In a study investigating exosome release during PEDV infection, we found that miRNA-328-3p is associated with PEDV infection [15]. Here, we also found that miRNA-328-3p can downregulate ZO-1 expression, though no direct evidence of miRNA-328-3p interacting with ZO-1 was found (results not shown). Interestingly, the downregulation of ZO-1 also impacts PEDV proliferation. Proteomic sequencing revealed that miRNA-328-3p downregulates nine proteins, including PLC-β1. We hypothesize that miRNA-328-3p regulation of ZO-1 may be linked to PLC-β1.

PLC proteins are typically involved in endothelial cell migration and angiogenesis and are associated with the regulation of TJ proteins. Previous studies have shown that inhibiting PLC-β can block LPA-induced claudin-7 expression in Caco-2 cells [25]. miRNA-328-3p may similarly reduce PLC-β1 expression, indirectly leading to decreased ZO-1 expression. Our data indicate a positive correlation between PLC-β1 and ZO-1 expression, suggesting that reduced PLC-β1 levels correspond to decreased ZO-1 levels. Furthermore, PLC-β1 does not directly affect ZO-1 expression; this effect may be mediated through PKC. PLC-β can regulate PKC expression via PLC-ε and DAG, and PKC activation directly enhances intestinal epithelial integrity by activating ZO-1 [26]. Interestingly, our data support that PLC-β1 can indirectly influence ZO-1 expression through PKC. Overall, the regulation of ZO-1 by miRNA-328-3p is complex, indicating that miRNA-328-3p is involved in PEDV-induced intestinal damage.

In summary, PEDV-induced miRNA-328-3p reduces PLC-β1 protein levels, and PLC-β1 influences ZO-1 expression through the PKC pathway. This may be one of the reasons for decreased ZO-1 expression post-PEDV infection, potentially affecting tight junctions and contributing to intestinal barrier damage. However, the mechanisms by which PEDV regulates miRNA-328-3p expression and the relationship between ZO-1 downregulation and reduced PEDV infection capability remain unclear, necessitating further research. PEDV infection is negatively correlated with ZO-1 expression, and ZO-1 expression simultaneously affects PEDV infection, which may indicate a complex interplay between PEDV and tight junction protein. In short, our study provides insights into miRNA-328-3p and TJ protein changes induced by PEDV infection and may help elucidate the mechanisms of PEDV-induced intestinal barrier damage.

## Supporting information

S1 Fig ZO-1 and PEDV are localized in the IPEC-J2 cells. (DOCX)
