# Supplementary material for: miRNA-328-3p regulates ZO-1 expression and inhibits PEDV proliferation via the PLC-β1-PKC pathway
Source: PLoS One. 2025 Jan 3;20(1):e0316074. doi: 10.1371/journal.pone.0316074 (PMC11698390; doi:10.1371/journal.pone.0316074)
Supplement: S1 Fig — (DOCX) [file pone.0316074.s001.docx]

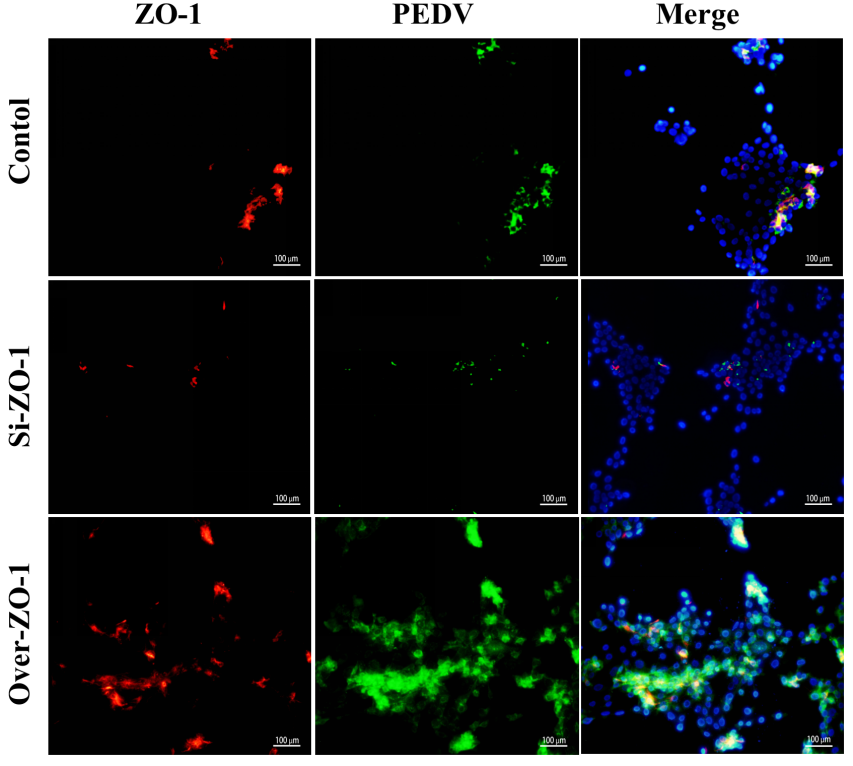


**Figure S1 ZO-1 and PEDV are localized in the IPEC-J2 cells**. After silencing or overexpressing the ZO-1 protein in IPEC-J2 cells, they are infected with PEDV, and representative confocal microscopy images are selected for observation. The red fluorescence corresponds to ZO-1, the green fluorescence to PEDV, and the blue fluorescence to the cell nucleus. Scale bars: 100 μm.
